# Early indicators and risk factors associated with mental health problems during COVID-19 quarantine: Is there a relationship with the number of confirmed cases and deaths?

**DOI:** 10.1177/0020764020966020

**Published:** 2020-10-15

**Authors:** Héctor Badellino, María Emilia Gobbo, Eduardo Torres, María Emilia Aschieri

**Affiliations:** 1 Faculty of Psychology, UCES University, San Francisco, Córdoba, Argentina; 2 CIECS (CONICET y UNC) y FCE-Universidad Nacional de Córdoba, Córdoba, Argentina; 3 School of Languages, Universidad Nacional de Córdoba, Córdoba, Argentina

**Keywords:** Anxiety, COVID-19, depression, quarantine, stress, sleep

## Abstract

**Background:**

On March 20 2020, the Argentine Ministry of Health enforced a mandatory quarantine throughout the country in response to the COVID-19 pandemic.

**Aims:**

The object of this study is to determine the initial impact on mental health of Argentine population, by measuring the prevalence of anxiety, depression, insomnia, and self-perceived stress and by determining the associated risk factors, and to analyze that impact in relation to the number of confirmed cases and deaths.

**Method:**

A cross-sectional survey was conducted through a digital questionnaire, which was completed by 1,985 respondents between March 29 and April 12. The prevalence of anxiety, depression, stress and insomnia was measured with the Generalized Anxiety Disorder-7 Scale (GAD-7), the 9-Item Patients Health Questionnaire (PHQ-9); the Perceived Stress Scale (PSS-10) and the Pittsburgh Sleep Quality Index (PSQI), respectively.

**Results:**

The 62.4% of the surveyed population reported signs of psychological distress. It was found that being a woman, being 18 to 27 years old, living with family members or a partner, smoking, and having a poor sleep quality were the significant risk factors.

**Conclusion:**

Despite the low number of COVID-19 confirmed cases and deaths at that time, a strong impact on mental health indicators was revealed. The authors of this study recommend the monitoring of the population at risk over time and early interventions in order to avoid long-lasting mental health problems.

## Introduction

In December 2019, the first cases of Severe Acute Respiratory Syndrome were reported in Wuhan (China), caused by a novel coronavirus (2019-nCoV), later called SARS-CoV2 (Huang et al., 2020). The disease rapidly spread around the world, bringing the World Health Organization (WHO) to declare the COVID-19 outbreak a pandemic on March 11 (World Health Organization, 2020). On March 3 Argentine authorities confirmed the first case of COVID-19 (Ministerio de Salud de la República Argentina, 2020a) and on March 20 they announced a number of prevention measures against the disease, including a mandatory quarantine (Gobierno de la República Argentina, 2020). The current study was started 9 days after mandatory confinement was ordered. By that time, there were 745 confirmed cases and 19 deaths in Argentina, and 634,835 confirmed cases and 29,891 deaths around the world (Ministerio de Salud de la República Argentina, 2020b).

Given the characteristics of the pandemic (high contagiousness, mortality, absence of effective treatments, no knowledge of disease evolution, etc.) (Huang et al., 2020; Zavascky & Falci, 2020), public health organisms determined that isolation and social distancing, which implied the confinement of the population and the interruption of all activity outside homes, were effective in preventing the virus dissemination, and hence, they would help to “flatten the curve of infections.” In preceding epidemics, those measures had a strong psychosocial and economic impact on inhabitants, communities and countries (Asmundson & Taylor, 2020; Taylor, 2019; Tull et al., 2020). Earlier studies demonstrated that during the course of isolation measures the levels of anxiety and fear to get sick or die increase, which are in turned increased by the excessive information spread on the media and the circulation of fake news (Rubin & Wessely, 2020).

In investigations carried out in China at the outset of the pandemic, it was detected that the general population, especially certain population groups (healthcare workers, students, and the elderly) showed high levels of anxiety, perceived stress, and poor sleep quality. (Cao et al., 2020; Li et al, 2020; Lima et al., 2020; Liu et al, 2020; Pappa et al., 2020; Qiu et al., 2020; Wang, Pan, Wan, Tan, Xu, Ho, et al., 2020; Wang, Pan, Wan, Tan, Xu, McIntyre, et al., 2020; Wang, Wang, et al., 2020; Wu & Wei, 2020; Xiao et al., 2020; Yang & Ma, 2020). Those studies demonstrated that women, students, and the elderly were seriously affected by high levels of anxiety and stress. Possibly, the most studied groups were healthcare workers (particularly female nurses) and students. These su bjects, especially the ones living in cities with a high prevalence of SARS-CoV2, showed more disturbances in their mood and sleep quality than the general population. (Cao et al., 2020; Chew et al, 2020; Li et al,2020; Pappa et al., 2020; Xiao et al., 2020). People who already had, prior to the pandemic, mental health disorders or addictions were also affected (Da et al., 2020; King et al., 2020; Mediouni et al., 2020; Ornell et al., 2020; Rhem et al., 2020). However, bibliography at the beginning of the current study was scarce. There is no bibliography about the impact of the pandemic on the Argentine population on search sites to date.

## Methods

### Study design and study population

We conducted this cross-sectional survey to determine the psychological impact of the COVID-19 pandemic on Argentine population by using an anonymous, voluntary online questionnaire. The online survey was made with a digital tool (Google Forms) and was spread on social networks (Facebook, Twitter, Instagram) and by email; it was administered from March 29 to April 12. Inclusion criteria were as follows: (a) be 18 years old or over, (b) live in Argentina. Exclusion criteria were as follows: suffer from (a) a previous mental disorder, (b) dyslexia.

First, participants were asked to provide their demographic and social information: age (age groups: 18–27, 28–39, 40–64, and 65 or older), sex, education level (primary, secondary or higher education), city of residence, quarantine compliance, size of dwelling, and way of living (living alone or with family, friends or partner). Next, they were asked to complete the questionnaires for measuring psychological distress and sleep quality.

### Tools to measure the impact on mental health and sleep quality

The following tools were used to measure the impact on mental health and sleep quality:

#### GAD-7 (Generalized Anxiety Disorder-7 items Scale)

Each question is given a value from 0 to 3. Possible answers were not at all sure (0), several days (1), over half the days (2) and nearly every day (3). The total test score can range from 0 to 21. The cut-off point is 10 (86.8% sensitivity and 93.4% specificity) and the level anxiety is classified as follows: Minimum (0–4), Mild (5–9), Moderate (10–14), and Severe (15–21) (Kroenke et al., 2007). Only respondents who reported moderate or severe anxiety were considered.

#### PHQ-9 (Patients Health Questionnaire 9-items)

This is an instrument to assess the presence and severity of depression symptoms. It consists of nine questions, each rated from 0 to 3. Possible answers were not at all sure (0), several days (1), over half the days (2), and nearly every day (3). The PHQ-9 scores are as follows: None (0–5), Mild (6–8), Moderate (9–14), and Severe (15–27). Only respondents who reported moderate or severe depression (9–27 points) were considered.

#### PSS-10 (Perceived Stress Scale)

This is an instrument to measure the level of perceived stress. It consists of 10 questions, each rated from 0 to 4. Possible answers were never (0), almost never (1), sometimes (2), fairly often (3), and very often (4). The cut-off point is 20, from which the level of perceived stress is considered to be high (Campo-Arias et al., 2009).

#### PSQI (Pittsburgh Sleep Quality Index)

This is a self-report questionnaire that contains nine questions (19 items) and it is used to measure sleep quality and sleep disturbances during the past month. In this case, following Liu et al., only the following four items of the index were selected to measure sleep quality: (a) How would you rate your sleep quality overall? (0. Very good; 1. Fairly good; 2. Fairly bad and 3. Very bad), (b) How often have you had trouble sleeping because you cannot get to sleep within 30 minutes? (0. Not during the past month; 1. Less than once a week; 2. Once or twice a week; 3. Three or more times a week), (c) How often have you had trouble sleeping because you wake up in the middle of the night or early morning? (0. Not during the past month; 1. Less than once a week; 2. Once or twice a week; 3. Three or more times a week), and (d) How many hours of actual sleep do you get at night? (Less than 5 hours per night; 6–7 hours per night; More than 7 hours per night) The use of some items has been seen in other studies since it has been proved that the use of isolated components for measuring subjective sleep quality highly correlates with the global PSQI score (Carpenter & Andrykowski, 1998). Responses assigned the two highest values in the scale and the response “less than 7 hours” for the last question were considered.

### Data analysis

The first step consisted in the creation of contingency tables with the descriptive analysis of the data among the independent variables versus the dependent variables in order to know, in general terms, the relationship between these variables (*t*-test). Then, multiple binary logistic regressions were performed to evaluate in each model the relationship between the dependent variables (Anxiety, Depression or Self-perceived Stress) and the independent variables, using a 95% confidence level to construct the intervals corresponding to Odds Ratios and to consider the statistical significance. *p*-value <.05 was considered to be significant. The software used was SPPS 9.0 (IBM SPSS Statistics, New York, United States).

### Ethical approval

A consent form was signed by all participants before they completed the survey. The study was approved by the Ethics Committee of Hospital Regional José Iturraspe of San Francisco city, Córdoba, Argentina.

## Results

### Prevalence and demographic characteristics

Out of a total of 2,051 collected surveys, 66 were excluded as they were responded by people not living in Argentina, leaving 1,985 valid surveys. The mean age of participants was 36.83 ± 14.41. Of all respondents, 75.9% (1,507) were women. Table 1 summarizes the demographic characteristics of the study population.

**Table 1.** Association between socio-demographic variables and anxiety, depression and stress level during the COVID-19 outbreak in Argentine population (*n*: 1,985).

Variables			Anxiety				Depression				Stress	
			Minimun	Mild	Moderate	Severe	None	Mild	Moderate	Severe	Non-significant	Significant
Sex	Female	1,505 (75.8)	647 (43)	597 (39.7)	204 (13.5)***	57 (3.8)	768 (51)	337 (22.4)	302 (20)**	98 (6.5)*	1,113 (74)	392 (26)***
	Male	480 (24.2)	288 (60)	153 (31.9)	29 (6.1)	10 (2.1)	293 (61)	102 (21.2)	67 (14)	18 (3.8)	417 (86.8)	63 (13.2)
Age	18–27 years old	695 (35)	262 (37.8)	292 (42.1)	103 (14.6)*	38 (5.5)	240 (34.6)	168 (24.2)	207 (29.7)***	80 (11.6)*	483 (69.5)	212 (30.5)***
	28–39 years old	471 (23.7)	214 (45.4)	198 (42)	45 (9.6)	14 (3)	257 (54.6)	119 (25.2)	80 (17.1)*	15 (3.2)	371 (78.7)	100 (21.3)
	40–65 years old	771 (38.9)	427 (55.4)	245 (31.8)	84 (10.8)	15 (2)	523 (67.8)	151 (19.6)	78 (10.2)	19 (2.5)	635 (82.4)	136 (17.6)
	over 65 years old	48 (2.4)	32 (66.7)	15 (31.3)	1 (2.1)	0 (0)	43 (89.6)	3 (6.3)	2 (4.2)	0 (0)	42 (87.5)	6 (12.5)
Quarantine compliance	Yes	1,666 (83.9)	776 (46,6)	648 (38.9)	189 (11.4)	52 (3.1)	868 (52.1)	371 (22.3)	324 (19.5)*	103 (6.2)	1,272 (76.4)	394 (23.6)
	No	319 (16.1)	158 (49.5)	102 (32)	44 (13.8)	15 (4.7)	194 (60.8)	69 (21.6)	43 (13.5)	13 (4.1)	259 (81.2)	60 (18.8)
Size of dwelling	Small	113 (5.7)	50 (44.2)	45 (39.8)	17 (15)	1 (0.9)	56 (49.6)	23 (20.4)	26 (23)	8 (7.1)	86 (76.1%)	27 (23.9)
	Big	1,872 (94.3)	884 (47.2)	706 (37.7)	216 (11.6)	66 (3.5)	1,005 (53.7)	417 (22.3)	341 (18.2)	108 (5.8)	1,444 (77.2)	427 (22.8)
Cohabitation	With others	1,757 (88.5)	798 (45.4)	678 (38.6)	215 (12.2)	66 (3.7)*	936 (53.2)	392 (22.3)	325 (18.5)	104 (5.9)	1,330 (75.7)	427 (24.3)***
	Alone	228 (11.5)	136 (59.6)	72 (31.6)	18 (7.9)	2 (0.9)	126 (55.3)	48 (21.1)	42 (18.4)	12 (5.3)	201 (88.2)	27 (11,8)
Education levelª	University	1,423 (71.7)	678 (47.6)	539 (37.9)	158 (11.1)	48 (3.4)	795 (55.8)	312 (22)	243 (17.1)*	73 (5.1)*	1,121 (78.8)	302 (21.2)**
	Non-university	562 (28.3)	256 (45.6)	212 (37.7)	75 (13.3)	19 (3.4)	268 (47.7)	128 (22.8)	123 (21.9)	43 (7.7)	410 (73)	152 (27)
Health worker	Yes	326 (16.4)	151 (46.5)	118 (36.3)	49 (14.8)	8 (2.5)	201 (61.5)	74 (22.8)	40 (12.3)**	11 (3.4)**	263 (80.7)	63 (19.3)
	No	1,659 (83.6)	783 (47.2)	632 (38.1)	185 (11.2)	59 (3.5)	861 (51.9)	366 (22.1)	327 (19.7)	105 (6.3)	1,268 (76.4)	391 (23.6)
Smoker	Yes	388 (19.5)	173 (44.6)	129 (33.2)	69 (17.8)***	17 (4.4)	174 (44.8)	83 (21.4)	99 (25.5)***	32 (8.2)*	283 (72.9)	105 (27.1)*
	No	1,597 (80.5)	761 (47.7)	621 (38.9)	164 (10.3)	50 (3.1)	888 (55.6)	357 (22.4)	268 (16.8)	84 (5.3)	1,248 (78.1)	349 (21.9)
Inability to sleep	Yes	1,023 (51.5)	353 (34.5)	452 (44.2)	164 (16)***	54 (5.3)***	383 (37.4)	271 (26.5)	275 (26.9)***	94 (9.2)***	700 (68.4)	323 (31.6)***
	No	962 (48.5)	582 (60.5)	298 (31)	69 (7.2)	13 (1.4)	679 (70.6)	169 (17.6)	92 (9.6)	22 (2.3)	831 (86.4)	131 (13.6)
Waking up in the middle of the night or early morning	Yes	1,078 (54.3)	375 (34.8)	465 (43.2)	180 (16.7)***	57 (5.3)***	451 (41.9)	275 (25.5)	256 (23.8)***	96 (8.8)***	753 (69.9)	325 (30.1)***
	No	907 (45.7)	559 (61.6)	285 (31.4)	53 (5.8)	10 (1.1)	610 (67.3)	165 (18.2)	111 (12.2)	21 (2.3)	778 (85.8)	129 (14.2)
Hours of sleep	7 or more	1,434 (72.2)	734 (51.2)	533 (37.2)	136 (9.5)	31 (2.2)	826 (57.6)	304 (21.2)	245 (17.1)	59 (4.1)	1,162 (81)	272 (19)
	Less than 7	551 (27.8)	200 (36.4)	217 (39.5)	97 (17.6)***	36 (6.5)***	235 (42.7)	136 (24.7)	122 (22.2)***	58 (10.4)***	369 (67)	182 (33)***
**Sleep quality**	Bad	459 (23.1)	119 (25,9)	182 (39.7)	113 (24.6)***	45 (9.8)***	122 (26.6)	123 (26.8)	139 (30.3)***	75 (16.3)***	268 (58.4)	191 (41.6)***
	Good	1,526 (76.9)	816 (53.4)	568 (37.2)	120 (7.9)	22 (1.4)	940 (61.6)	317 (20.8)	228 (15)	41 (2.7)	1,263 (82.8)	263 (17.2)

The education level was transformed into two variables (High school or below and University and postgraduate). Size of dwelling was transformed into two groups (big house or apartment with balcony or garden vs small house or apartment without balcony or garden).

* *p* ⩽ .05. **.01. ***p ⩽ .001.

As regards age, 695 (35%) respondents were between 18 and 27 years old; 471 (23.7) were between 28 and 39 years old; 771 (38.9%) were between 40 and 64 years old and 48 (2.4%) were 65 years old or older. Of all participants, 326 (16.4%) were health workers, 228 (11.5%) lived alone, 319 (16.1%) did not quarantine for some reason, 388 (19.5%) smoked and 1,423 (71.7%) were studying for a degree or had already finished their higher education.

As regards sleep quality, 1,023 (51.1%) participants had trouble in getting to sleep within 30 minutes, 1,078 (54.3%) participants woke up in the middle of the night or early morning, 551 (27.8%) participants slept less than 7 hours and 459 (23.1%) participants rated their sleep quality as fairly bad or very bad.

### Factors related to anxiety

The number of respondents suffering from anxiety was 1,050 (52.8%), of which 750 (37.8%) reported mild anxiety, 233 (11.7%) reported moderate anxiety, and 67 (3.3%) reported severe anxiety. In analyzing the factors related to anxiety, only participants who scored moderate or severe anxiety (300 = 15.1%) were considered. In those 300 respondents, the prevalence of moderate to severe anxiety is significantly higher in women (261 = 17.3%) than in men (39 = 8.1%) (Table 1).

As regards age, there was a significant prevalence of moderate (103 = 14.6%) or severe (38 = 5.5%) anxiety in young people between 18 and 27 years old, compared to the 28 to 39 age group (45 = 9.6% and 14 = 3%, respectively), the 40 to 64 age group (84 = 10.8% and 15 = 2%, respectively) and the 65 or over age group (1 = 2.1% and 0%, respectively). Respondents who lived alone showed lower levels of moderate to severe anxiety (20 = 8.8%) than those who lived with family members, partners or friends (281 = 15.9%). The difference in severe anxiety was statistically significant. Smokers showed a significantly high level of anxiety (86 = 22.2%), compared to non-smokers (214 = 13.4%). There was no statistically significant difference in the prevalence of anxiety in health personnel, nor in relation to the size of dwelling, level of education or quarantine compliance.

Later, the risk factors of moderate and severe anxiety were analyzed (Table 2). The female sex was significantly associated with moderate to severe anxiety: OR: 2.121; 95% Confidence Interval (95% CI: 1.455–3.092) *p*: .000. Respondents between 18 and 27 years old were at significantly increased risk: OR: 8.205 (95% CI: 1.097–61.378) *p*: .04. The levels of anxiety were significantly higher in people living with a partner, children o friends. OR: 1.829 (95% CI: 1.091–3.067) *p*: .022. The health personnel were also at high risk of suffering from anxiety. OR: 1.595 (95% CI: 1.101–2.309) *p*: .014 and smokers exhibited similar figures: OR: 1.832 (95% CI: 1.337–2.506) *p*: .000.

**Table 2.** Association between significant socio-demografic variables and risk factors for moderate to severe anxiety, depression and significant stress during the COVID-19 outbreak in Argentine population (*n*: 1,985).

Variables	Anxiety			Depression			Stress		
	OR	95% CI	*p*	OR	95%CI	*p*	OR	95%CI	*p*
Female sex (Ref. male sex)	**2.121**	**1.455–3.092**	**0.000**	**1.666**	**1.236–2.246**	**0.001**	**2.151**	**1.584–2.921**	**.000**
18–27 years of age (Ref. 65 years or over)	**8.205**	**1.097–61.378**	**0.04**	**12.978**	**3.037–55.452**	**0.001**	2.087	0.844–5.161	.111
28–39 years of age (Ref. 65 years or over)	4.626	0.615–34.934	0.138	**4.858**	**1.127–20.95**	**0.034**	1.487	0.595–3.719	.396
40–64 years of age (Ref. 65 years or over)	5.045	0.674–37.765	0.115	2.964	0.69–12.738	0.144	1.219	0.492–3.019	.669
Health worker (Ref. non-health worker)	**1.595**	**1.101–2.309**	**0.014**	0.838	0.581–1.208	0.344	0.981	0.703–1.37	.914
Living with others (Ref. living alone)	**1.829**	**1.091–3.067**	**0.022**	0.744	0.508–1.09	0.129	**2.245**	**1.439–3.502**	**.000**
Smoker (Ref. non-smoker)	**1.832**	**1.337–2.506**	**0.000**	**1.73**	**1.304–2.299**	**0.000**	1.277	0.965–1.689	.087
Inability to get to sleep within 30 minutes (Ref. no)	**1.372**	**1.005–1.873**	**0.047**	**2.336**	**1.792–3.04**	**0.000**	**1.759**	**1.362–2.273**	**.000**
Waking up in the middle of the night (Ref. no)	**2.315**	**1.661–3.226**	**0.000**	**1.908**	**1.462–2.494**	**0.000**	**1.672**	**1.29–2.169**	**.000**
Bad sleep quality (Ref. good)	**3.226**	**2.404–4.310**	**0.000**	**2.66**	**2.041–3.472**	**0.000**	**2.174**	**1.681–2.187**	**.000**
Size of dwelling (Ref. big)	1.159	0.640–2.096	0.626	1.085	0.662–1.779	0.747	1.195	0.73**–**1.956	.48
Level of education (Ref. University)	1.024	0.753–1.392	0.878	1.033	0.795–1.342	0.808	1.215	0.943–1.566	.132
Duration of sleep (Ref. 7 hours or more)	0.986	0.714–1.362	0.933	0.778	0.596–1.014	0.064	1.042	0.801–1.355	.76

The bold indicates the significance of the results.

The other demographic variables weren’t found to be risk factors for moderate or severe anxiety: being 28 years or over, size of dwelling, level of education, and sleep hours.

### Factors related to depression

Of all participants, 924 (46.5%) showed signs of depression, of which 439 (22.1%) scored mild depression, 369 (18.6%) scored moderate depression, and 116 (5.8%) scored severe depression. In analyzing the factors related to depression, only respondents who scored moderate or severe depression were considered (485 = 24.4%). In those 485 respondents, the prevalence of moderate or severe depression is significantly higher in women (400 = 26.6%) than in men (85 = 17.7%) (Table 1).

There was a significant prevalence of moderate (29.7%) and severe (11.6%) depression in young people between 18 to 27 years old, compared to the 28 to 39 age group (17.1% and 3.2% respectively), the 40 to 64 age group (10.2% and 2.5%, respectively) and the 65 or over age group (4.2% and 0%, respectively). A statistically greater difference in moderate depression was also found in the 28 to 39 age group, compared to the groups over 40 years. Respondents who complied with quarantine (1,666) had a higher prevalence of moderate depression (324 = 19.5%) than those who for some reason did not (319), in whom the prevalence of moderate depression was lower (43 = 13.5%). Smokers showed a significantly high level of moderate or severe depression (131 = 33.7%), compared to non-smokers (352 = 22.1%). Health workers showed a significantly lower prevalence of moderate or severe depression (51 = 15.7%) than non-health workers (432 = 26%). Respondents who had already finished college had also a significantly lower prevalence of depression (316 = 22.2%) than those who had only completed primary or secondary education (166 = 29.6%). There was no statistically significant difference in the prevalence of depression in relation to the size of dwelling nor to the fact of living alone or with others.

The risk factors that contributed to moderate or severe depression are listed below (Table 2): being a women OR: 1.666 (95% CI: 1.236–2.246) *p*: .001; being 18 to 27 years of age: OR: 12.978 (95% CI: 3.037–55.452) *p*: .001; being 28 to 39 years of age: OR: 4.858 (95% CI: 1.127–20.95) *p*: .034; smoking: OR: 1.73 (95% CI: 1.304–2.299) *p*: .000. The following variables do not constitute risk factors for moderate or severe depression: adults between 40 and 65 years and over, health workers, living alone or with others, size of dwelling, and education level.

### Factors related to stress

When we analyzed the factors related to self-perceived stress (Table 1), 455 (22.9%) of the participants reported high levels of stress. In those 455 respondents, the prevalence of moderate or severe stress was significantly higher in women (392 = 26%) than in men (63 = 13.2%). Young people between 18 and 27 years old showed significantly higher levels of stress (212 = 30.5%) than the 27 to 39 age group, the 40 to 64 age group (136 = 17.6%) and the 65 or over age group. (6 = 12.5%). Respondents who lived with others reported significantly higher levels of stress (427 = 24.3%) than those who lived alone (27 = 11.8%). Smokers showed higher levels of stress (105 = 27.1%) than non-smokers (349 = 21.9%). Respondents who had finished university had a lower level of stress (302 = 21.2) than those who had only completed primary or secondary education (152 = 27%). There was no statistically significant difference in relation to the size of dwelling, quarantine compliance, or health personnel.

The risk factors that contributed to stress are listed below (Table 2): being a women: OR: 2.151 (95% CI: 1.584–2.921) *p*: .000 and living with others: OR: 2.245 (95% CI: 1.439–3.502) *p*: .000.

### Sleep quality and psychological impact

Regarding sleep, there is a significant relationship between moderate or severe anxiety levels and difficulty for falling asleep, waking up in the middle of the night, sleeping less than 7 hours, and perception of poor sleep quality. All differences were statistically significant, which shows a connection between sleep quality and high levels of anxiety during the day.

Participants who had trouble in getting to sleep within 30 minutes were at high risk for moderate to severe anxiety: OR: 1.372 (95% CI: 1.005–1.873) *p*: .047; respondents who woke up in the middle of the night or early morning: OR: 2.315 (95% CI: 1.661–3.226) *p*: .000 and those who assessed their sleep quality as bad o very bad: OR: 3.226 (95% CI: 2.404–4.310) *p*: .000.

A statistically significant relationship was also found between moderate or severe depression and difficulty for falling asleep, waking up in the middle of the night or early in the morning, sleeping less than 7 hours, and perception of poor sleep quality.

As with anxiety, those who had trouble in getting to sleep within 30 minutes were at high risk for moderate or severe depression: OR 2.336 (95% CI: 1.792–3.04) *p*: .000; respondents who woke up in the middle of the night or early morning: OR: 1.908 (95% CI: 1.462–2.494) *p*: .000 and those who assessed their sleep quality as bad or very bad: OR: 2.66 (95% CI: 2.041–3.472) *p*: .000.

As with anxiety and depression, a statistically significant relationship was also found between high stress levels and difficulty for falling asleep, waking up in the middle of the night or early in the morning, sleeping less than 7 hours, and perception of a poor sleep quality.

The risk for stress was significantly increased among respondents who had trouble in getting to sleep within 30 minutes: OR 1.759 (95% CI: 1.362–2.273) *p*: .000; respondents who woke up in the middle of the night or early morning: OR: 1.672 (95% CI: 1.29–2.169) *p*: .000 and those who assessed their sleep quality as bad o very bad: OR: 2.174 (95% CI: 1.681–2.187) *p*: .000.

There was not a significant relationship of risk between sleep hours and anxiety, depression, or stress.

## Discussion

This is the first report in literature that shows the psychological impact of social isolation measures in the context of a pandemic in the Argentine population. So far, the scientific evidence available worldwide is scarce, with most publications originating in China.

This cross-sectional study was begun 9 days after the mandatory quarantine was enforced, and despite that short time, signs of psychological impact were found in 62.4% of all respondents, of which 15.1% reported moderate or severe anxiety, 24.4% reported moderate or severe depression, and 22.9% showed high stress levels. There were 745 confirmed cases and 19 deaths at the time the study was initiated (03/29/2020).

The disease appeared in Argentina later than in China and Europe; the media and social networks greatly contributed to disseminating the idea (and fear) that we were about to enter a stage never seen in the history of our country.

The level of impact on mental health demonstrated in this study was higher than the level shown by Wang, Pan, Wan, Tan, Xu, Ho, et al. (2020) during the initial stage of the epidemic in China (When that study was started on January 31, 2020, 9,723 cases and 213 were reported in China) probably because the 2-month period between both studies contributed to increasing the fear of the pandemic, that was starting to spread in Argentina. At that moment, the economic context of our country was characterized by high levels of unemployment and poverty, and the health system was experiencing structural and functional difficulties. But the three parameters were even higher than those obtained in a study conducted in Spain only 2 weeks before our investigation (Ozamiz-Etxebarria et al., 2020), although in both cases different instruments were used to measure anxiety, depression and stress. When the Ozamiz-Etxabarria et al. (2020) study was initiated (03/14/2020), 4,231 confirmed cases and 1,268 deaths were reported on that day in Spain. Our findings revealed a lower impact than the outcomes obtained in Italy (Mazza et al., 2020) on March 18, 2020, with 31,506 confirmed cases and 2,503 deaths.

Our hypothesis is that despite the low number of confirmed cases and deaths in our country, the strong impact on the mental health of the general population could be linked to the following factors: massive dissemination of information about what was happening in China and Europe, several travelers having to return to the country from their holiday destinations, unfavorable social and economic conditions and inadequate public health system.

Our study demonstrates that the impact of the social, preventive, and mandatory confinement on mental health is higher in women than in men. It is worth mentioning that most women began to work from home (teleworking), while they were also in charge of looking after their children and helping them with their online school homework. All this contributed to increase their stress levels since their daily habits were dramatically changed during quarantine. These findings adhere to previous international epidemiological studies that evidenced that women were at higher risk of depression (Lim et al., 2020) and post-traumatic stress (Liu et al., 2020).

Young people showed a higher risk of having anxiety, depression and stress, compared to older adults, especially the 18 to 27 age group, who reported a significantly high risk of anxiety and depression. Given that the majority of the youngest respondents (18–27 years old) were university students, it is possible that some factors (adaptation to a new educational context with online classes, fear that the new reality could negatively affect their academic progression, isolation and scarce contact with peers) may contribute to increase the levels of anxiety, depression and stress, at an age in which those mental disorders are prone to develop. Previous studies carried out on university students in China show similar results (Cao et al., 2020; Li et al., 2020), although bibliography is scarce.

In relation to the low prevalence of anxiety, depression, and stress in older adults (65 years or older), although this is the age group at the highest risk for developing complications and with the highest mortality rate (Zavascky & Falci, 2020), they are probably more adapted to living in isolation and having little social life. On the other hand, the number of respondents of that age group was low due to the need to use digital technology. This fact does not allow for assuming a response accounting for the low prevalence and risk of those mental disorders.

Contrary to what was expected, respondents who lived alone had significantly lower levels of anxiety and stress than those who lived with family, partner, or friends. Bibliography accounting for this phenomenon was not found, but it is possible that people living alone are more adapted to isolation, whereas the permanent, extended contact inside home during the confinement may increase the levels of stress and anxiety in people living with a partner, children, etc.

The health personnel surveyed had a higher risk of anxiety than the general population, though their levels of depression and stress were not significant. This is contrary to what was found in the meta-analysis published by Pappa et al. (2020), which shows a greater impact on the mental health of healthcare workers than on the general population. The low psychological impact on this group may be linked to the fact that, at the time this study was conducted, the number of COVID-19 confirmed cases at national level was low, with areas of the country without confirmed cases.

Smokers showed a significant risk of anxiety and depression, and an increased but not significant risk of stress. The link between smoking and anxiety-depression has been evidenced in several publications (Fluharty et al., 2017), although it is not possible to establish a clear causal relationship or to know which of the two factors precedes the other.

A significant relationship was also found between bad sleep quality (which includes difficulty for falling asleep, waking up in the middle of the night or early in the morning, and the self-rated overall sleep quality) and the prevalence of anxiety, depression, and stress. There was no significant relationship with the number of hours the respondents slept. The relationship between mental health indicators and poor sleep quality has been evidenced in numerous studies conducted on the general population and on risk groups such as health professionals or students (Pappa et al., 2020; Wu & Wei, 2020; Xiao et al., 2020; Yang & Ma, 2020).

No association could be established between the mental disorders studied and size of the dwelling and educational level.

The triad—being a woman, being young, and living with others—seems to be an interesting risk group that we believe should be studied as quarantine extends.

Our study has limitations. In the first place, the cross-sectional nature of the study does not allow an interpretation for causality, but it shows in a short time window a picture of what is happening at a psychological level in a new and unpredictable reality. Secondly, since this study was disseminated through social networks and it required the use of digital technology, participation was limited since a large part of the population doesn’t have access to digital technology or lacks the knowledge of how to use it. These limitations are common in studies of this nature, but we believe that by knowing a snapshot of the psychological reality, measures can be taken to effectively prevent the long-term effects that a situation of this nature can cause.
